# Automated surgical planning in spring-assisted sagittal craniosynostosis correction using finite element analysis and machine learning

**DOI:** 10.1371/journal.pone.0294879

**Published:** 2023-11-28

**Authors:** Jenson Jacob, Selim Bozkurt

**Affiliations:** Ulster University, School of Engineering, Belfast, United Kingdom; Universiti Sains Malaysia, MALAYSIA

## Abstract

Sagittal synostosis is a condition caused by the fused sagittal suture and results in a narrowed skull in infants. Spring-assisted cranioplasty is a correction technique used to expand skulls with sagittal craniosynostosis by placing compressed springs on the skull before six months of age. Proposed methods for surgical planning in spring-assisted sagittal craniosynostosis correction provide information only about the skull anatomy or require iterative finite element simulations. Therefore, the selection of surgical parameters such as spring dimensions and osteotomy sizes may remain unclear and spring-assisted cranioplasty may yield sub-optimal surgical results. The aim of this study is to develop the architectural structure of an automated tool to predict post-operative surgical outcomes in sagittal craniosynostosis correction with spring-assisted cranioplasty using machine learning and finite element analyses. Six different machine learning algorithms were tested using a finite element model which simulated a combination of various mechanical and geometric properties of the calvarium, osteotomy sizes, spring characteristics, and spring implantation positions. Also, a statistical shape model representing an average sagittal craniosynostosis calvarium in 5-month-old patients was used to assess the machine learning algorithms. XGBoost algorithm predicted post-operative cephalic index in spring-assisted sagittal craniosynostosis correction with high accuracy. Finite element simulations confirmed the prediction of the XGBoost algorithm. The presented architectural structure can be used to develop a tool to predict the post-operative cephalic index in spring-assisted cranioplasty in patients with sagittal craniosynostosis can be used to automate surgical planning and improve post-operative surgical outcomes in spring-assisted cranioplasty.

## 1. Introduction

Sagittal synostosis is the most common type of cranial anomaly comprising around 50% of craniosynostosis conditions [1]. It is caused by the fused sagittal suture and results in a narrowed skull [2]. In sagittal synostosis, bossing in the forehead occurs due to longitudinal skull growth and narrowing [3, 4].

Endoscopic methods in sagittal craniosynostosis are used to correct skull deformities over time therefore they rely on skull growth [1]. Endoscopic strip craniectomy can be used in patients with sagittal craniosynostosis between 4 and 6 months with subsequent helmet therapy [5] to improve cosmetic and functional outcomes. The cephalic index may remain suboptimal in comparison to open surgical techniques due to rapid skull growth [5]. However, open surgical methods to correct sagittal synostosis are preferred after 6 months old [6]. Spring-assisted cranioplasty is another method being used to correct sagittal craniosynostosis, however, the outcome of both endoscopic and spring-assisted methods remain similar [7] whilst spring-assisted cranioplasty corrects the skull over time, therefore, the outcome of this procedure depends also on the skull growth [8].

Computational methods such as finite element analysis have been proposed to predict the outcome of craniosynostosis correction to improve surgical outcomes. For instance, Borghi et al. [9] used numerical simulations to evaluate spring-assisted cranioplasty in a patient-specific sagittal craniosynostosis model. Bozkurt et al. [10] utilised finite element analyses to evaluate different options in the correction of unicoronal craniosynostosis. An in-silico modelling platform was developed and used to predict spring-assisted posterior vault expansion [11]. Computational modelling was also used to predict outcomes of spring-assisted cranioplasty in lambdoid craniosynostosis [12]. Different correction techniques for sagittal craniosynostosis were also compared using computational simulations in patient-specific skull models [13]. Although finite element analyses can simulate displacements for different correction techniques they also require validation or iterative simulations for optimal surgical settings. Therefore, the efficiency of finite element models predicting surgical outcomes in craniosynostosis may be compromised and must be improved. Machine learning methods offer opportunities as diagnostic and surgical planning tools in medicine [14]. For instance, Knoops et al. [15] developed a machine learning framework for automated diagnosis in plastic surgery. The use of machine learning algorithms has also been proposed in neurosurgery as preoperative surgical planning tools [16]. Machine learning methods have also been used to evaluate the effect of metopic severity on the aesthetic outcome of fronto-orbital advancement in metopic craniosynostosis [17]. A similar approach can also be implemented in finite element models to increase the efficiency of the simulations and predict surgical outcomes more accurately in the correction of sagittal craniosynostosis. Moreover, novel automated surgical planning tools based on machine learning and computational simulations can improve the outcome of personalised treatment where there is limited data for rare diseases such as sagittal craniosynostosis. Therefore, the aim of this study is to develop the architectural structure of an automated tool to predict post-operative surgical outcomes in sagittal craniosynostosis correction with spring-assisted cranioplasty using machine learning and finite element analyses.

## 2. Materials and methods

There is no human data in this manuscript. A statistical shape model for representing average skull shape in sagittal craniosynostosis patients was used to develop finite element models and the statistical shape model can be found on Zenodo, an open-access database [18].

**Fig 1:**
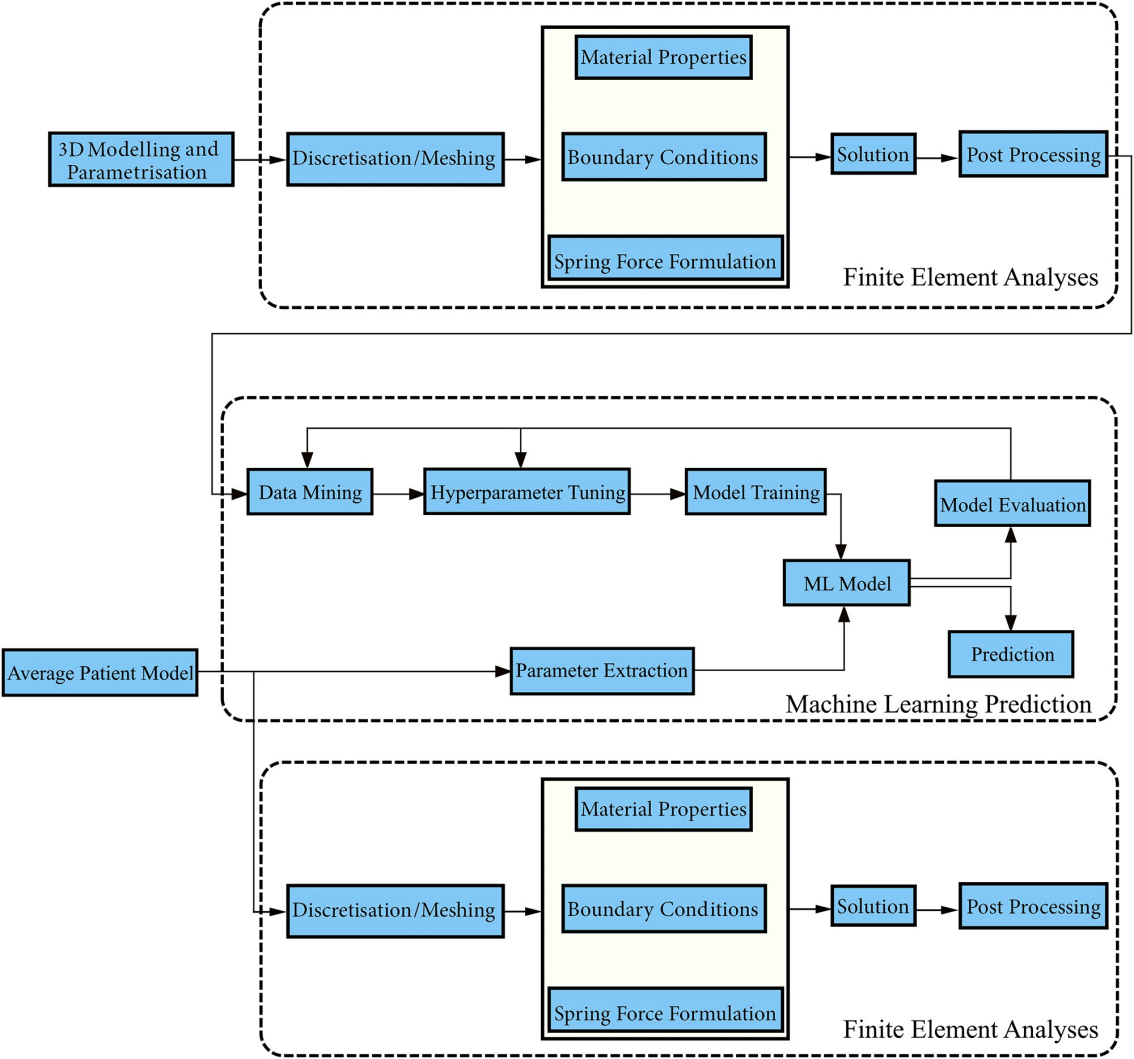
The workflow of the developed modelling and Machine Learning (ML) architectural structure.

Six different machine learning algorithms were tested using a finite element model which simulated a combination of various mechanical and geometric properties of the calvarium, osteotomy sizes, spring characteristics, and spring implantation positions. The finite element model was developed using a parametric 3D solid model which represents a calvarium with sagittal craniosynostosis. The workflow of the developed modelling and machine learning architectural structure is given in Fig 1.

### 2.1. Parametric skull model and finite element simulations

The developed architectural structure starts with 3D modelling and parametrisation of a calvarial model. This model is then meshed and mechanical properties, boundary conditions and spring forces are defined. The solution is obtained through finite element analyses and the results are processed. The processed results were used to tune hyperparameters and train the machine learning algorithms. The post-operative cephalic index was predicted using an average template which was obtained through statistical shape modelling and the prediction was validated using finite element analyses.

A 3D calvarial model was developed considering average length, width, height, and anterior and posterior curvatures for infant skulls around 5-month-old with sagittal craniosynostosis [19, 20] using Solidworks 2021 (Dassault Systèmes, Vélizy-Villacoublay, France). The 3D calvarial model is given as a S1 File. The cephalic index (CI) in the developed calvarial model was calculated as given below.


$$\text{CI}=\frac{\text{Skull}\;\;\text{Width}}{\text{Skull}\;\;\text{Length}}\times 100$$ (1)

The length, width and height of the skull model were modelled as 165 mm, 116 mm and 87 mm considering data given by Heutinck et al. [20]. The cephalic index in the skull model was 0.70. Hexahedral quadratic elements were used to discrestise the created calvarial geometry in MSC Marc 2022 (Hexagon, Stockholm, Sweden). Coronal and lambdoid sutures and anterior fontanelle were created considering the anatomical features of a skull affected by sagittal craniosynostosis around 5 months of age [21] in MSC Marc 2022. Around two hundred ten finite element simulations were performed to obtain training data for the machine learning algorithms using the developed finite element analysis. Mechanical properties of the bony parts in the skull, skull thickness, width and length of the osteotomy, position, and number of the implanted springs, and spring diameter were selected as the variables in the simulations.

The elastic modulus of parietal bones in skulls with sagittal craniosynostosis changes between 1000 MPa and 4500 MPa in children around 5 months old [22]. However, relatively low values for elastic modulus bone elastic modulus have been reported in the literature [23, 24]. Therefore, the range of the bone elastic modulus was changed between 100 MPa and 3000 MPa in the simulations. Values such as 0.22 or 0.28 have been reported for Poisson’s ratio of the bones in infants [25, 26]. Therefore, the range of bone Poisson’s ratio was defined between 0.2 and 0.3 in the simulations. The thickness of parietal bones changes between 1.5 mm and 3 mm in children around 6 months old [27] whereas the mean bone thickness in parietal bones and frontal bones in infants between 0 to 6 months old is around 3.4 mm and 3.7 mm respectively [28]. Therefore, the range of the skull thickness was set between 2 mm to 4 mm in the simulations. The width of the osteotomy in Borghi et al. [9] was 20 mm whereas 10 mm wide osteotomies have also been performed [29]. Therefore, the width of osteotomy sizes varied between 10 mm to 20 mm in the simulations. Osteotomy is performed between coronal and lambdoid sutures to insert the springs [30]. The length of osteotomy in the simulations is defined using the distance from the sutures. The distance between the sutures and osteotomy varied between 0 mm to 40 mm in the simulations. Two or three springs were simulated considering the number of the implanted springs in patients [31]. Springs are positioned 30 to 40 mm from the sutures [29] whereas a 10 mm spring distance from the sutures has also been used in clinics [32]. Therefore, the range for the distance of the springs from the sutures was defined between 10 mm and 40 mm. Three different spring characteristics depending on the wire diameter were simulated as described by Borghi et al. [33]. Also, the 3D skull model was scaled to simulate different calvaria sizes. The range of the skull length and width were changed between 153 mm and 170 mm and 107 mm and 120 mm [8, 20] respectively. The range of the variables in the finite element simulations which were used to train and test the machine learning algorithms is given in Table 1.

**Table 1:** Range of the variables in the finite element simulations which were used to train and test the machine learning algorithms. E and ν represent Elastic modulus and Poisson’s ratio of the bones, t_skull_ represents the skull thickness, w_ost_ and l_ost_ represent the width of the osteotomy and the distance between the sutures and osteotomy, n_spring_ is the number of the springs implanted in the skull, x_spring_ is the distance between the springs and both ends of the sutures, l_skull_ and w_skull_ represent skull length and width.

Parameter	Range	References
E [Mpa]	1000–4500	[22–24]
v	0.2–0.3	[25,26]
t_skull_ [mm	2–4	[27,28]
w_ost_ [mm]	10–20	[9,29]
I_ost_ [mm]	0–40	[30]
l_skull_ [mm]	153–170	[8,20]
w_skull_ [mm]	107–120	[8,20]
x_spring_ [mm]	10–40	[29,32]
n_spring_	2–3	[31]

The variables within the defined range were generated randomly to simulate the training data. Elastic modulus and Poisson ratio of sutures and anterior fontanelle were set to 16 MPa and 0.49 in all the simulations [9, 12]. Fixed displacement boundary conditions were used at the base of the skull model.

### 2.2. Machine learning algorithms

Linear regression, support vector regression, decision tree, random forest, gradient boosting, and XGBoost machine learning algorithms were used to predict the post-operative cephalic index in spring-assisted sagittal craniosynostosis correction.

Linear regression uses a linear relationship between the input variables (x_1_, x_2_,…,x_n_) and the output variable (ŷ) [34].


$$\hat{y}=\beta_{0}+\beta_{1}x_{1}+\beta_{2}x_{2}+\ldots +\beta_{n}x_{n}$$ (2)

Support Vector Regression utilises support vectors to approximate the function that best fits the training data. It is the summation of the product of support vector coefficients (αᵢ) and the kernel function (K) applied to the input vectors (xᵢ) and the test instance (x), along with a bias term (b) [35].


$$\hat{y}=\sum_{i}^{N}\left(\alpha_{i}\times K({\text{x}}_{i},\text{x})\right)+b$$ (3)

Decision trees partition the feature space into regions and assign a constant value (cᵢ) to each region. The predicted value (ŷ) is determined by averaging the values of the training instances falling into the corresponding region [36].


$$\hat{y}=\sum_{i}^{N}\left(c_{i}\right)/N$$ (4)

Random Forest combines multiple decision trees to make predictions. Each tree produces a predicted value (ŷᵢ) based on a subset of features and training instances. The final prediction (ŷ) is obtained by averaging the predicted values of all the trees [37].


$$\hat{y}=1/N\sum_{i}^{N}\left({\hat{y}}_{i}\right)$$ (5)

Gradient Boosting builds an ensemble of typically decision trees sequentially. Each learner tries to correct the mistakes made by the previous learners. The final prediction (ŷ) is the sum of the predictions of all the learners [38]. eXtreme Gradient Boosting (XGBoost) is an optimised implementation of gradient boosting. It follows a similar approach as gradient boosting but incorporates additional regularisation techniques to enhance performance [39]. Output (ŷ) in gradient boosting and XGBoost is described in the same way.


$$\hat{y}=\sum_{i}^{N}\left(F_{i}(x)\right)$$ (6)

The machine learning algorithms were trained using data obtained from the finite element simulations, and the hyperparameters were tuned accordingly to ensure that the algorithms would perform well in test data. Hyperopt package in Python was used to tune hyperparameters [40]. A search space that specifies the range and type of hyperparameters to optimise was defined. An adaptive Tree of Parzen Estimators was used to find the optimum hyperparameters in the search space. Through multiple iterations, Hyperopt refined the search space and converged towards the optimal hyperparameters for the machine learning task. The performance of the machine learning algorithms was evaluated using root mean squared error and coefficient of determination (R^2^). Scikit-learn library in Python was used to run the machine learning algorithms. In total two hundred and twelve finite element analyses were performed to obtain training and test data. Simulation results from one hundred seventy simulations were used to train the machine learning algorithms whereas forty-two simulation results were used as test data. The parameters extracted in the machine learning algorithms were used as features using the training set. The test data were the target variables results obtained from the finite element simulations.

### 2.3. Validation of machine learning predictions via a statistical skull model and finite element simulations

**Fig 2:**
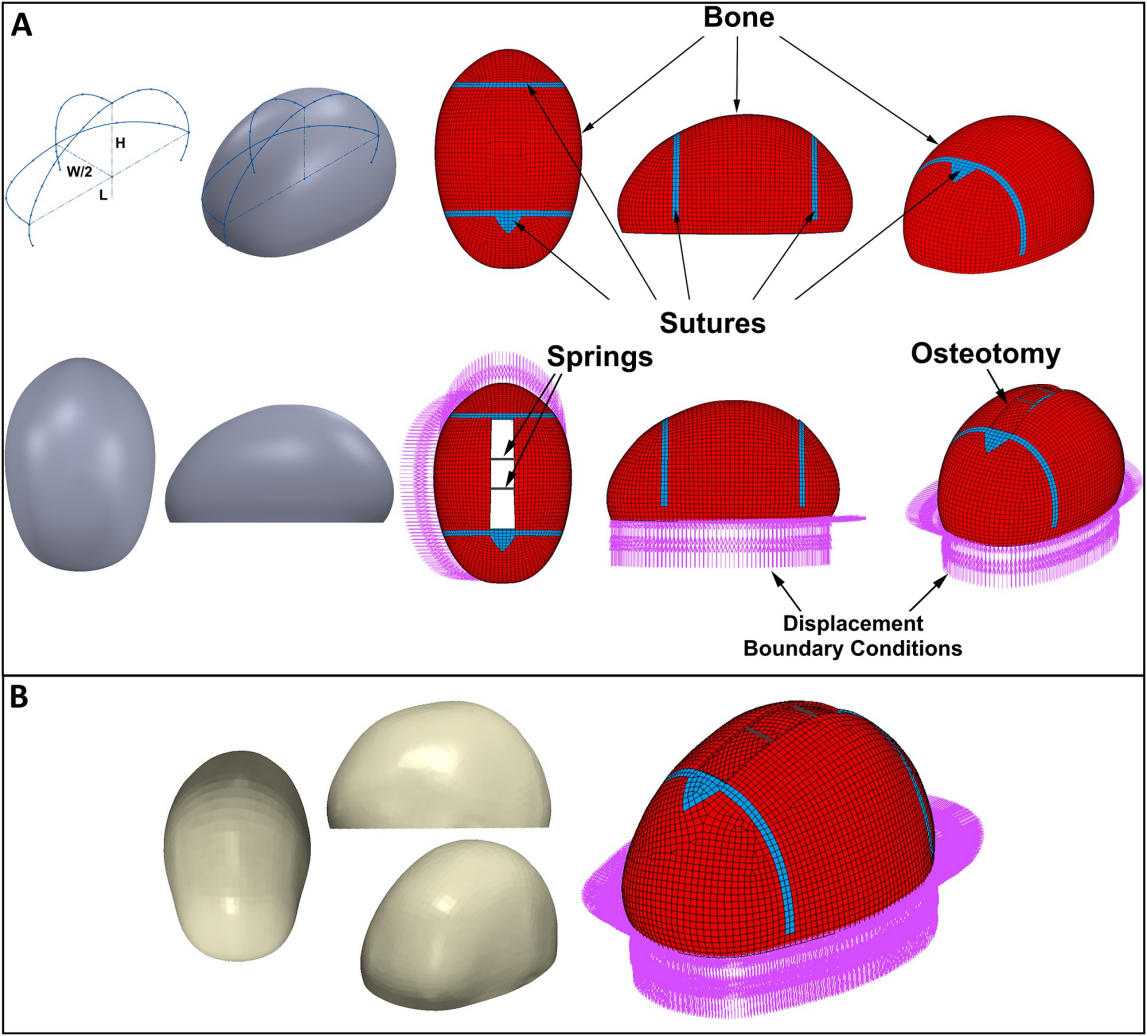
**A)** The geometric calvarium model, finite element model for the pre-operative skull, and a finite element model with osteotomy and springs (L, W, and H represent length, width, and height), **B)** The statistical shape model for sagittal craniosynostosis and the finite element model with osteotomy and springs to test the machine learning algorithms.

A statistical shape model for sagittal craniosynostosis available on Zenodo open access database [18, 41] was used as test data to verify the performance of the machine learning algorithms with finite element models. The average age of the patients in this model was around 5 months old. The length, width, and height of the skull model were around 160mm, 114 mm, and 99 mm resulting 0.71 cephalic index. Elastic modulus (E) and Poisson Ratio (ν) of the bone and sutures were 41 MPa and 16 MPa and 0.22 and 0.49 respectively [9]. The skull thickness was 2 mm. An osteotomy was modelled by removing the elements from the coronal suture to lambdoid suture resulting in an around 97 mm long cut whereas the width of the osteotomy was 20 mm. Two 1.2 mm diameter springs were positioned at 34 mm distance from the sutures. Compressed spring forces were modelled considering the data given by Borghi et al. [33]. The post-operative cephalic index was used to compare the prediction in the machine learning algorithms and the finite element model simulating spring-assisted cranioplasty in the statistical shape model for sagittal craniosynostosis. The geometric calvarium model, finite element model for the pre-operative skull, and finite element model with osteotomy and springs and the statistical shape model for sagittal craniosynostosis and the finite element model with osteotomy and springs to validate the machine learning algorithms are given in Fig 2.

### 2.4. Mesh sensitivity analysis

**Fig 3:**
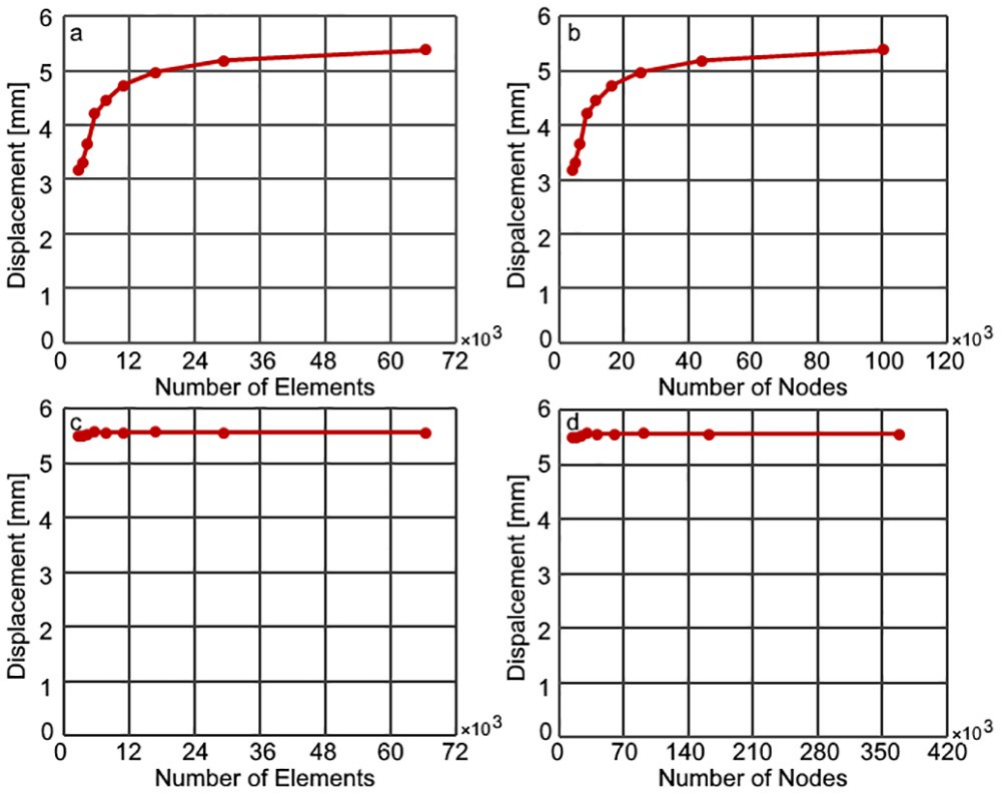
Mesh sensitivity in the finite element model a) number of elements in the simulations with hexahedral linear elements b) number of nodes in the simulations with hexahedral linear elements, c) number of elements in the simulations with hexahedral quadratic elements d) number of nodes in the simulations with hexahedral quadratic elements.

Around 7500 hexahedral quadratic elements with 42000 nodes were used in the simulations after evaluating the mesh dependence in the model. The finite element simulations to evaluate mesh sensitivity were done using 421 MPa and 0.22 elastic modulus and Poisson’s ratio in the bones, 16 MPa, and 0.49 elastic modulus and Poisson’s ratio in the sutures. The skull thickness was 2 mm. The width of the osteotomy was 20 mm whereas the osteotomy was performed from the coronal suture to the lambdoid suture. Two springs made of 1.2 mm wire were positioned at 34 mm distance from the sutures. Results for the mesh sensitivity test are given in Fig 3.

## 3. Results

**Fig 4:**
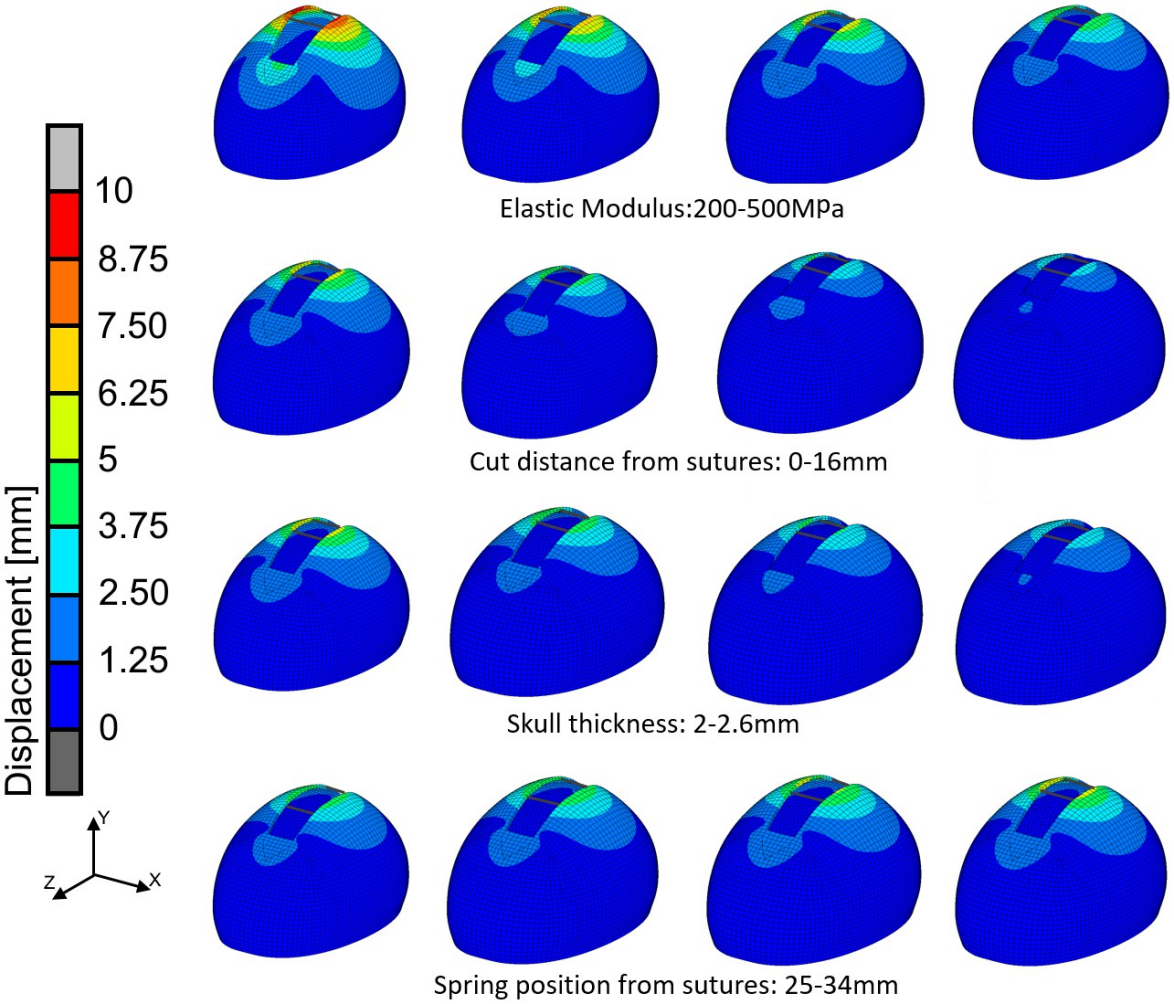
Displacement map for a range of elastic modulus, osteotomy distances from the sutures, skull thickness, and spring positions.

Displacement map for a range of elastic modulus, osteotomy distances from the sutures, skull thickness, and spring positions for 20 mm osteotomy and two springs made of 1.2 mm wire are given in Fig 4.

**Fig 5:**
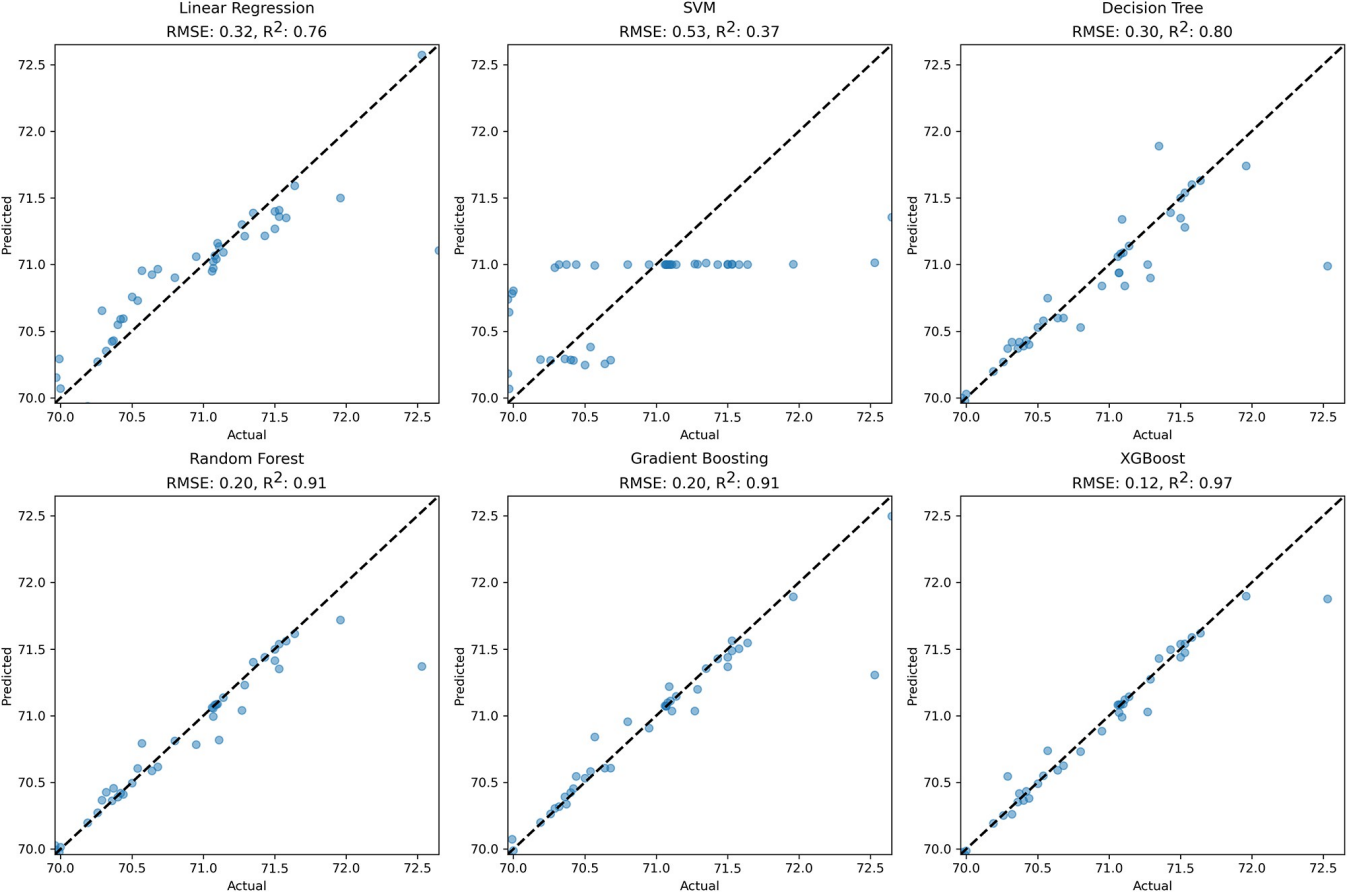
Correlations between the simulated and predicted post-operative cephalic index for the machine learning algorithms (RMSE and R^2^ represent root mean square error and coefficient of determination).

Maximal displacement in the skull model decreased with the increasing elastic modulus. Longer osteotomy sizes resulted in higher displacements in the skull model. Also, relatively low skull thickness allowed higher displacement in the skull. Positioning springs closer to the sutures also resulted in relatively large displacements in the skull model. Correlations between the simulated and predicted post-operative cephalic index for each machine learning algorithm are given in Fig 5.

**Fig 6:**
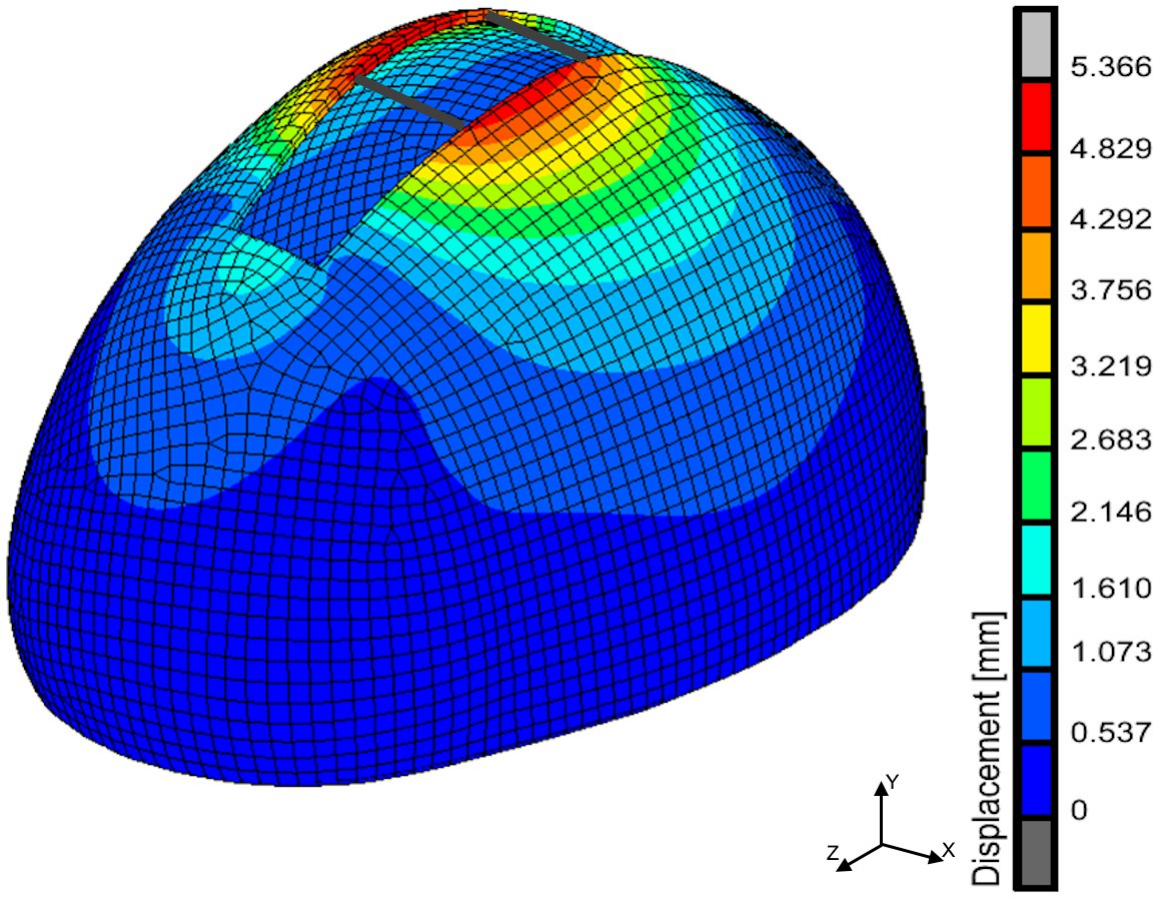
The displacement map in the finite element model simulating spring-assisted cranioplasty in the statistical shape model for sagittal craniosynostosis.

Root mean square errors in Linear Regression, Support Vector Regression and Decision Tree algorithms were around 0.20 or higher whereas the coefficients of determination (R^2^) in these algorithms were lower than 0.90. Root mean square errors and coefficients of determination (R^2^) in Random Forest, Gradient Boosting and XGBoost were 0.20, 0.20 and 0.12 and 0.91, 0.91 and 0.97 respectively The displacement map in the finite element model simulating spring-assisted cranioplasty in the statistical shape model for sagittal craniosynostosis is given in Fig 6.

**Fig 7:**
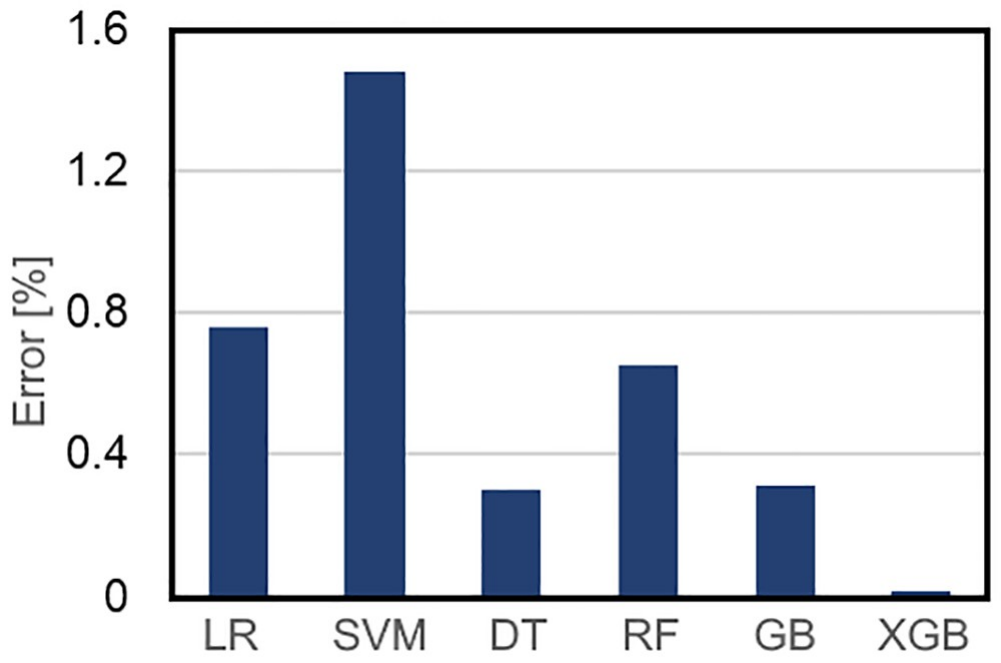
The percentages of error for the predicted post-operative cephalic indexes by each machine learning algorithm (LR, SVM, DT, RF, GB, and XGB represent linear regression, support vector regression, decision tree, random forest, gradient boosting, and extended gradient boosting respectively).

The maximal displacement in the statistical skull model after spring implantation was around 5.3 mm. Also, the post-operative cephalic index was around 0.723 in the finite element model simulating spring-assisted cranioplasty in the statistical skull model for sagittal craniosynostosis. The percentages of error for the predicted post-operative cephalic indexes by each machine learning algorithm are given in Fig 7.

The percentage of the error was the highest in the Support Vector Regression algorithm whereas the XGBoost algorithm predicted the post-operative cephalic in the statistical shape model index with minimal error. Errors in the cephalic index predicted by the other machine learning algorithms were also higher than the error in the cephalic index predicted by the XGBoost algorithm.

## 4. Discussion

In this study, the performance of six different machine learning algorithms which were used to predict post-operative cephalic index after spring-assisted correction of sagittal craniosynostosis was evaluated. A parametric 3D solid model representing calvarium was used to simulate spring-assisted correction of sagittal craniosynostosis utilising finite element analysis. The finite element simulations were performed to generate training data for the machine learning algorithms. A statistical shape model of sagittal craniosynostosis was used as the test model and the predicted post-operative cephalic index by each algorithm was validated using finite element analysis.

The parametric solid model used in this study allowed changing the geometric parameters such as thickness within the anatomical range for patient age considered [27, 28] and osteotomy length and width as in the reported literature [9, 29]. Craniosynostosis is a rare disease with different phenotypes [42], therefore, the parametric model was also used to populate training and testing data by considering the mechanical properties of infant skull affected by sagittal craniosynostosis until six months of age [22]. Our analysis showed that the XGBoost algorithm predicts the post-operative cephalic index with a very low error outperforming the other machine learning algorithms tested in this study. Error in the predicted post-operative cephalic index was relatively high in the other tested machine learning algorithms.

In this study, the outcome of the spring-assisted correction in the cranial model was evaluated using the cephalic index, therefore, machine learning algorithms were trained to predict the cephalic index. Total and compartmental cranial volumes have also been suggested to quantify head shape and outcomes after cranioplasty in sagittal craniosynostosis [43]. The reason for evaluating the cephalic index was because the aesthetic success of surgical intervention in sagittal synostosis is measured using the cephalic index [44]. The average post-operative cephalic index in sagittal synostosis is between 70% and 72% [8]. Clinical data show that the cephalic index increases between 3% and 9% after removal of the springs in patients with sagittal synostosis [45]. However, the increase in the cephalic index depends on the cranial bone properties as well as the thickness of the bones [45]. Another study shows that one-year post-operative outcomes show that the cephalic index increases around 3% after spring-assisted correction of sagittal craniosynostosis [46]. The post-operative cephalic index values in the finite element simulations which were used to test the machine learning 70% and 72.5%. In the patient-specific skull model, the cephalic index increased by more than 1% after spring insertion. However, the simulated results show the immediate post-operative effect of the spring forces on the cephalic index. The relatively high increase in the cephalic index reported in clinical data shows the effect of skull growth and spring forces over time.

Proposed tools to plan patient-specific surgeries in craniosynostosis correction include tools such as computed tomography imaging [47], rapid prototyping [48, 49] or finite element simulations [50]. Nonetheless, finite element analyses require iterative simulations to find optimal surgical parameters such as spring positions or the size of the osteotomy whereas 3D-printed templates do provide information about the post-operative surgical outcome. The presented architectural structure with a machine learning algorithm has the potential to overcome the aforementioned challenges. Spring-assisted cranioplasty in sagittal craniosynostosis can be planned after training and testing the machine learning algorithm. The machine learning algorithm can predict surgical outcomes such as the cephalic index. Finite element analyses can be performed to simulate displacements on the skull and validate the prediction following the simulations from the machine learning algorithm. The proposed workflow will reduce the number iterative finite element simulations whilst automating the surgical planning in spring assisted sagittal craniosynostosis correction.

In this study, six different machine learning algorithms were used. Each algorithm has shortfalls. For instance, although linear regression is a simple and interpretable model, it assumes a linear relationship between input features and the target variable and may not capture complex non-linear patterns in the data, potentially leading to reduced predictive accuracy [51]. Selecting appropriate hyperparameters can be challenging, and improper tuning may result in suboptimal performance in Support Vector regression [52]. Decision trees are prone to overfitting, especially when the tree depth is not properly controlled. They may create overly complex models that do not generalise well to unseen data [36]. Although random forests mitigate the overfitting issue of decision trees by aggregating multiple trees, they can be computationally expensive. Training numerous decision trees may lead to longer training times and increased memory usage [53]. Gradient boosting methods are susceptible to outliers in the data. Outliers can have a significant impact on model performance, requiring robust preprocessing techniques [54]. XGBoost is known for its robustness and efficiency. However, like other ensemble methods, it may require careful tuning of hyperparameters, such as the learning rate and tree depth, to achieve optimal results [39]. XGBoost may be a suitable algorithm to train and utilise to plan spring-assisted sagittal craniosynostosis correction and will likely perform better than other approaches in a future clinical study. The results were not validated using clinical data and validation of the results which is a limitation of the study will be a future task.

## 5. Conclusion

The presented architectural structure to predict the post-operative cephalic index in spring-assisted cranioplasty in patients with sagittal craniosynostosis has the potential to automate surgical planning. An automated surgical planning tool will improve post-operative surgical outcomes in spring-assisted cranioplasty.

## Supporting information

S1 File The 3D parametric CAD model used in the simulations. (ZIP)
